# Analysis of the Human Kinome Using Methods Including Fold Recognition Reveals Two Novel Kinases

**DOI:** 10.1371/journal.pone.0001597

**Published:** 2008-02-13

**Authors:** Kristine M. Briedis, Ayelet Starr, Philip E. Bourne

**Affiliations:** 1 Bioinformatics Program, University of California San Diego, La Jolla, California, United States of America; 2 Department of Bioengineering, University of California San Diego, La Jolla, California, United States of America; 3 Skaggs School of Pharmacy and Pharmaceutical Sciences, University of California San Diego, La Jolla, California, United States of America; University of Queensland, Australia

## Abstract

**Background:**

Protein sequence similarity is a commonly used criterion for inferring the unknown function of a protein from a protein of known function. However, proteins can diverge significantly over time such that sequence similarity is difficult, if not impossible, to find. In some cases, a structural similarity remains over long evolutionary time scales and once detected can be used to predict function.

**Methodology/Principal Findings:**

Here we employed a high-throughput approach to assign structural and functional annotation to the human proteome, focusing on the collection of human protein kinases, the human kinome. We compared human protein sequences to a library of domains from known structures using WU-BLAST, PSI-BLAST, and 123D. This approach utilized both sequence comparison and fold recognition methods. The resulting set of potential protein kinases was cross-checked against previously identified human protein kinases, and analyzed for conserved kinase motifs.

**Conclusions/Significance:**

We demonstrate that our structure-based method can be used to identify both typical and atypical human protein kinases. We also identify two potentially novel kinases that contain an interesting combination of kinase and acyl-CoA dehydrogenase domains.

## Introduction

Most proteome-wide functional annotation focuses on sequence similarity, however, this ignores valuable information that protein structure can provide–an important consideration in the era of structural genomics when many more protein structures are becoming available [1]. In some cases, the sequence between two proteins has diverged too far to find any significant sequence similarity with current methods, but a structural similarity can still be seen [2]–[4]. For example, Hon *et al.* crystallized the aminoglycoside phosphotransferase APH(3′)-IIIa and found a surprising homology to eukaryotic protein kinases (ePKs) [5]. About half of the sequence folded into a structure typical of ePKs, despite a very low sequence identity. The major structural differences were found in the area of the protein that determined substrate specificity [5]. Likewise, Holm and Sander found two glucosyltransferases that shared less than 10% sequence identity, but still contained strong structural similarities that indicated evolutionary relatedness [6]. These two examples illustrate that the structures of proteins can reveal surprising similarities that are undetected by sequence identity alone. Notwithstanding, one must be cautious in assigning relatedness based on structural similarity alone. It is possible for two proteins with a similar structure to function in different ways. For example, lysozyme and α-lactalbumin have similar structures and a 40% sequence identity, but differ in function [7]. It is also possible for proteins to arrive at a similar structure through convergent rather than divergent evolution. Subtilisin and chymotrypsin are serine endopeptidases that share a catalytic triad, but no other sequence or fold similarity [7].

We have established a high-throughput approach to provide accurate structure and functional annotation termed the Encyclopedia of Life (EOL) [8], based on the desire to annotate a large number of sequenced proteomes. EOL uses a pipeline approach termed the integrated Genome Annotation Pipeline (iGAP), which we have applied in examining the set of human kinases, the human kinome, in an attempt to uncover distant homologs not previously seen.

**Figure 1:**
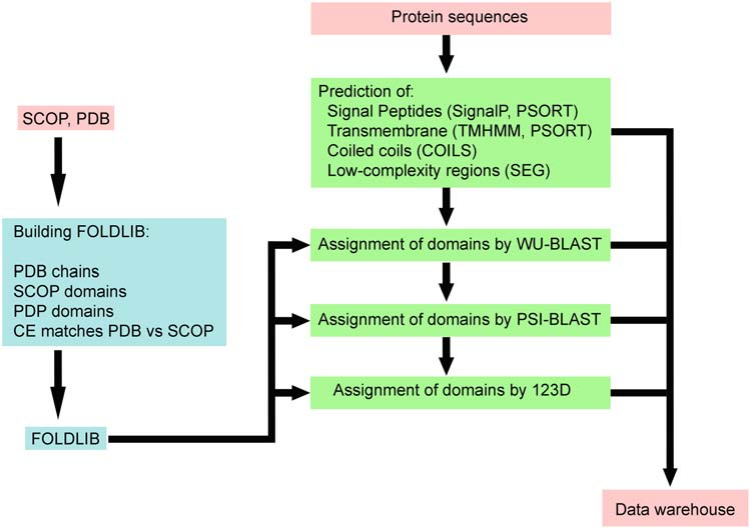
iGAP annotation pipeline. Diagram of the iGAP pipeline. Protein sequences are compared to a domain library using WU-BLAST, PSI-BLAST, and 123D.

iGAP (**Figure 1** **)** compares already identified protein sequences from whole proteomes against a comprehensive structure fold library (FOLDLIB). The fold library was built from a combination of Protein Data Bank (PDB) protein chains [9] and protein domains defined by SCOP [10] and PDP [11]. SCOP domain sequences were filtered at 90% identity. Since there is a delay between protein structures being added to the PDB and classified by SCOP, PDB chains were clustered at 90% identity, parsed with PDP, and added to the SCOP domains to generate a more complete library. The collection of SCOP, PDP and PDB sequences were then clustered at 90% identity to determine the final FOLDLIB composition [8].

The core of the pipeline consists of tools that search for sequence and fold similarity, including the sequence comparison programs WU-BLAST [12] and PSI-BLAST [13], and the threading program 123D [8], [14]. Protein sequences from completed proteomes were first compared to FOLDLIB using WU-BLAST. Then, PSI-BLAST profiles were generated for each input protein sequence using three iterations and a default H-value of 1e-06. Lastly, the protein sequences were compared to FOLDLIB using the fold recognition program 123D [8].

The result is a set of putative structure and function assignments including a novel statistical measure of reliability (Shindyalov *et al.* unpublished). Reliability is defined using a consensus approach with SCOP as a benchmark. Using a test set of non-redundant SCOP folds, Shindyalov *et al.* counted the number of consistently and inconsistently predicted assignments by WU-BLAST for each target sequence. The hits were binned by E-value and the specificity was averaged over all values in the bin, resulting in a reliability assignment. Reliability is defined as the number of positions with consistent predictions divided by the total number of positions having two or more hits to the same SCOP fold.

Using this method, it is found that the probability of traditional E-value assignments being correct varies between proteomes since they are not random, and indeed are not random in different ways. For example, using WU-BLAST to assign SCOP folds to proteomes, to reach a level of 1 error per 1000 annotations, one must use an E-value cutoff of 1×10^−8^ for *Arabidopsis thaliana* but only 1×10^−2^ for *Caenorhabditis elegans*. EOL individually benchmarks every genome and assigns a reliability index that can be used to compare different genomes. The reliability index is set by determining the E-values required for a sequence to be consistently identified with a fold and binning the hits by E-value. The resultant reliability index is termed A through E and corresponds to 99.9%, 99%, 90%, 50%, and 10% specificity, respectively [8].

We utilized this pipeline to characterize the collection of human protein kinases. Eukaryotic protein kinases (ePKs) regulate signal transduction reactions in the cell, influencing many processes including metabolism, apoptosis and transcription [15].

The collection of kinases has previously been defined by several groups including Cheek *et al*. [16] and Manning *et al*. [15]. Cheek *et al.* searched multiple species for all enzymes that catalyze the transfer of an ATP terminal phosphate group, while Manning *et al*. focused on both typical and atypical protein kinases in humans. Atypical protein kinases (aPKs) were defined by Manning *et al.* as proteins that have weak sequence similarity to the ePKs, but still have protein kinase activity.

Since our study focuses on the human protein kinase superfamily, we compared our results with that of Manning *et al.* [15]. They published the “complete” human kinome paper in 2002 based on homologies detected using Hidden Markov Models (HMMs). HMMs were developed by Manning *et al.* for the ePK family and the PIKK, RIO, ABC1, PDK, and alpha kinase atypical families. The HMMs were used to search against Genbank, SwissProt, dbEST, Celera human genome, Incyte LifeSeqGold, and internal SUGEN and Pharmacia sequence databases. Full-length gene predictions were determined for putative kinase hits, and confirmed in most cases by cDNA cloning [15].

Our approach differs in several ways. By including the threading program 123D, we incorporate fold recognition along with sequence similarity, possibly leading to the identification of more distant homologs. We also searched Ensembl's [17] draft assembly 34 v19.34.a.1 of the human genome, which presumably differs from the genome draft used by Manning *et al.* in 2001–2002.

Utilizing Hidden Markov Models (HMMs) along with EST and cDNA data, Manning *et al.* found 518 human protein kinases. This accounts for almost 2% of all human genes, and makes protein kinases one of the largest eukaryotic gene families [15]. Most human kinases contain a eukaryotic protein kinase (ePK) catalytic domain. This catalytic domain shows remarkable conservation, specifically with respect to critical residues and motifs, as previously described by Hanks and Hunter [18]. However, the HMM method employed by Manning *et al.* is only one approach to identifying specific protein families across a whole proteome. We thus compared the human kinome as classified by the EOL pipeline to the Manning set. We determined that our method performs well in classifying the kinome and we present here two putative novel kinases.

## Results and Discussion

Overall, the human kinase set identified here by EOL agreed with the set of kinases found by Manning *et al.* In addition, we analyzed 153 potential novel protein kinase sequences (selected as described in the Methods section) using Pfam [19] and found 44 contained an assignment for either an ePK or atypical kinase domain. Based on these Pfam results, our sequences were classified into the following groups (followed by the sequence count in parentheses): choline/ethanolamine kinase (5), fructosamine kinase (2), protein kinase (20), PI3_PI4 kinase (17) and not kinase (109) (See Supplementary Tables S1 and S2 for data).

Most of the differences between our human kinome and that found by Manning *et al.* can be attributed to analyzing a different draft of the human genome. Only one kinase exists in both Manning *et al.'s* human kinome and our Ensembl human genome draft that EOL did not identify (LRRK2 UniProt:Q5S2007 [20]). Upon further investigation, it was discovered that the Ensembl LRRK2 protein was only 400 amino acids long in our draft, and was missing the protein kinase domain. Ensembl lengthened the LRRK2 sequence in a subsequent draft to 2527 amino acids, including the protein kinase domain. Ten other protein kinases from the Manning *et al.* kinome match proteins in our set at a lower score than our cutoff for mapping, probably due to using slightly different gene predictions and data sets. These ten proteins, upon closer inspection, were manually mapped to the Manning *et al.* kinome. For example, ENSP00000330379 has a 98% local sequence identity to EphA10 in Manning *et al.'s* human kinome, but is 462 amino acids shorter. It is annotated in Ensembl as EphA10 precursor. The ten proteins, along with reasons for their poor mapping, are described in further detail in Supplementary Table S3.

Some of the kinases identified by EOL are from protein families that are part of the protein kinase-like SCOP superfamily (d.144.1), but are not classified in the “protein kinases, catalytic subunit” family. This includes the atypical kinase families actin-fragmin kinases, MHCK/EF2 kinases, phosphoinositide 3-kinases, choline kinases, aminoglycoside phosphotransferases, and the RIO1-like kinases [10]. Some of these EOL kinases were not present in Manning *et al.'s* set, but were already deposited and identified in NCBI's [21] Non-Redundant database (NR) as kinases. In an effort to pinpoint the source of differences, we looked at the methods used by Manning *et al.* to classify the sequences. Manning *et al.'s* paper states that they developed Hidden Markov Models (HMMs) for some of the atypical families, including PIKK, RIO, ABC1, PDK, and alpha kinase [15]. In comparison, the EOL search included only those atypical kinases present in the ePK superfamily, as defined by SCOP (d.144.1) [10]. Thus, it is not surprising that the EOL human kinome contains a different set of atypical kinases than Manning *et al.'s* kinome. For example, to the best of our knowledge Manning's group did not build an HMM to look for choline/ethanolamine kinases. EOL's human kinome, however, correctly classified five such proteins (SCOP family d.144.1.8) in the human proteome.

Here we focus on two particularly interesting potential kinases that were classified by Ensembl as acyl-CoA dehydrogenase family members. A BLAST search against NR showed these proteins to be ACAD10 [UniProt: Q6JQN1; Ensembl: ENSP00000325137] and ACAD11 [UniProt:Q709F0; Ensembl:ENSP00000264990].

**Figure 2:**
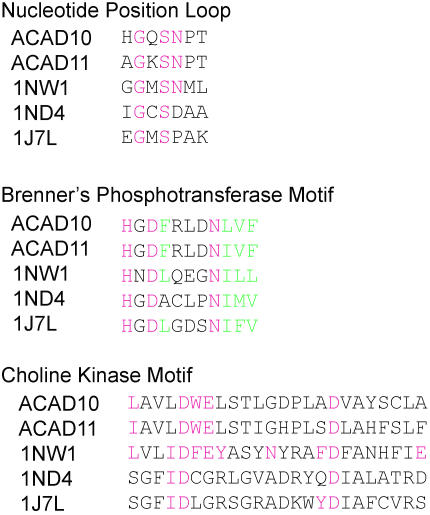
Conserved kinase motifs in ACAD10 and ACAD11. ACAD10 and ACAD11 contain conserved kinase motifs such as the nucleotide position loop, a phosphotransferase motif, and part of a choline kinase motif. Residues in pink are highly conserved; residues in green are commonly large hydrophobic amino acids. ACAD10 and ACAD11 are aligned with the choline kinase 1NW1 and aminoglycoside phosphotransferases 1ND4 and 1J7L for comparison.

ACAD10 has been previously identified as being involved in the β-oxidation of fatty acids [22]. EOL recognized the acyl-CoA dehydrogenase domain, but also assigned a kinase domain as part of the sequence. 123D produced the strongest kinase hit, with a weaker hit from PSI-BLAST. **Figure 2** shows an alignment of the protein to common kinase motifs. Clearly, the nucleotide position loop and Brenner's phosphotransferase motif [23] are well conserved. Less well conserved is the choline kinase motif. It is interesting to note, however, that some of the most critical functional residues of choline kinases as identified by Yuan *et al.* are conserved [24].

ACAD11 is 279 amino acids shorter than ACAD10, and has a similar arrangement of acyl-CoA dehydrogenase and kinase domains. The difference in length is mostly attributable to a hydrolase domain that is present in ACAD10, but not ACAD11. A BLAST alignment between ACAD10 and ACAD11 shared a 46% sequence identity overall (excluding the hydrolase domain), and a 48% sequence identity in the kinase domain. At the time of our initial study, the protein corresponding to ACAD11 in Ensembl did not contain a kinase domain. However, it has since been lengthened in a subsequent release and appears to contain a kinase domain with similar features to ACAD10, as shown in **Figure 2** (see Supplementary Figure S1 for a longer alignment).

The kinase domains of ACAD10 and ACAD11 appear to be most similar to a choline kinase or an aminoglycoside phosphotransferase (APH) domain. The similarity between the APH and choline kinase families was previously noted by Scheeff and Bourne [25] in a study of the structural evolution of the protein kinase-like superfamily. Structural analysis revealed conservation in their C-terminal subdomains that was not observed to exist in other kinase families [25]. EOL, Pfam, and Superfamily [26] annotate the protein kinase domains of ACAD10 and ACAD11 as an APH domain with higher confidence than a choline kinase domain, however, the aforementioned similarities to the choline kinase motif are intriguing.

Choline kinases phosphorylate choline to produce phophocholine [27]. This pathway eventually produces phosphatidylcholine, a component of cell membranes. Choline kinase is a particularly important atypical kinase as it has been shown to play a role in several types of cancer. Over-activity of choline kinase and increased concentrations of phosphocholine have been identified in breast cancer cells [28]. Increased phosphocholine levels have also been reported in prostate and brain tumors [29].

Aminoglycoside phosphotransferases (APHs) are also an interesting atypical kinase family, present in bacteria. As previously mentioned, Hon *et al.* revealed a surprising structural similarity between APH and eukaryotic protein kinases (ePK) [5]. APHs have been implicated in antibiotic resistance. They phosphorylate aminoglycoside hydroxyl groups. In bacteria this can result in inactivation of aminoglycoside antibiotics such as kanamycin and gentamicin. However, APHs have also been shown to phosphorylate some ePK substrates. Daigle *et al.* demonstrated that two APHs had the ability to phosphorylate some Ser/Thr protein kinase substrates, though at a slower rate than aminoglycoside phosphorylation [30]. This could perhaps offer an explanation as to how a kinase domain with similarities to APHs would function in eukaryotes.

**Figure 3:**
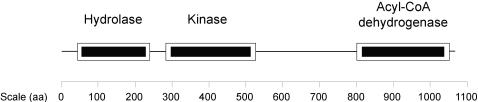
Domains identified in ACAD10. iGAP identified hydrolase, kinase, and acyl-CoA dehydrogenase domains in ACAD10.

The domain arrangement of ACAD10 shown in **Figure 3** was the only human protein identified as such in the Superfamily database [26]. Superfamily and Pfam found proteins with the same domain structure in *Mus musculus* (mouse), *Caenorhabditis elegans* (worm), *Caenorhabditis briggsae* (worm), *Bos taurus* (cow), *Ciona intestinalis* (sea squirt), *Macaca mulatta* (rhesus monkey), *Monodelphis domestica* (opossum) and *Pan troglodytes* (chimp) [19], [26]. Similar proteins also exist in bacteria [31].

In conclusion, we have utilized both sequence and structure-based tools to annotate the human kinome. We were successful in identifying both ePK and atypical kinases. We were particularly intrigued by ACAD10 and ACAD11, which contain acyl-CoA dehydrogenase and apparent kinase domains. The cellular function of such a combination of domains and the level of kinase activity for these proteins remains to be determined.

## Materials and Methods

Assembly 34 v19.34.a.1 of the Ensembl [17] human genome draft was run through iGAP [8], including WU-BLAST [12], PSI-BLAST [13], and the threading program 123D [14]. The subset predicted to contain the protein kinase superfamily was selected for further study. Protein kinase domains are generally 250–300 amino acids in length [18]. Thus, our set of candidate proteins was filtered to exclude near-identical sequences and those shorter than 200 amino acids to exclude proteins that despite a short sequence or structural similarity cannot contain a full, active kinase domain. Since it was unknown at the beginning of the study how sensitive iGAP would be in identifying full kinase domains, we selected proteins with a predicted kinase domain of 120 amino acids (roughly half the length of a typical protein kinase domain) or greater for further study. To ensure we didn't miss any abnormally short kinases, we also included any proteins that did not meet the above criteria, but appeared to contain at least two conserved subdomains from Hanks and Hunter's ePK domain analysis [18].

The proteins found were mapped to the Manning *et al.'s* human kinome using BLAST at a 90% sequence identity cutoff point. This strict threshold was set so proteins were not erroneously mapped to each other. However, it was done with the understanding that given human genome draft changes, some proteins may fall below this identity threshold that should be considered equivalent to each other.

Of the remaining 324 potentially unique proteins, 234 were selected that matched to a kinase domain by 123D, in hopes of exploiting any distant structural similarities that would be overlooked when considering sequence alone. Many of these predictions were at a lower reliability and were deemed false positives. These false positives likely share some structural, but not functional, similarity to the kinase fold. Sequences of “A” or “B” reliability were analyzed for conserved kinase domain motifs and blasted against NCBI's NR database [21]. Including the aforementioned sequences that contained Hanks and Hunter ePK subdomains [18], our final data set consisted of 153 sequences (Table S1).

## Supporting Information

Table S1 Set of possible protein kinases, as identified by EOL. 153 proteins identified by EOL as possible protein kinases were analyzed in depth for conserved kinase motifs. This table contains the Ensembl id, the reliability of the kinase match, the method that determined the match, the PDB ID of the kinase matched, and author comments.
(0.06 MB XLS)

Table S2 20 Protein Kinase Matches. This table contains details regarding the 20 protein kinase Pfam matches. The table includes the Ensembl ID, the closest protein kinase mapping, and author comments.
(0.02 MB XLS)

Table S3 Reclassified Ensembl Proteins. This table describes Ensembl IDs that showed poor mapping to Sugen kinases and had to be manually verified. The table contains the Ensembl ID, the kinase name, and the reason why the protein had to be mapped manually.
(0.02 MB XLS)

Figure S1 Kinase Domain Alignment of APH and Choline Kinase Proteins. This is a ClustalW alignment of sample choline kinase and aminoglycoside phosphotransferase domains with ACAD10 and ACAD11. Accession numbers are included in sequence names.
(0.04 MB DOC)
