# Supplementary material for: Analysis of the Human Kinome Using Methods Including Fold Recognition Reveals Two Novel Kinases
Source: PLoS One. 2008 Feb 13;3(2):e1597. doi: 10.1371/journal.pone.0001597 (PMC2223070; doi:10.1371/journal.pone.0001597)
Supplement: Supplementary file 1 [file pone.0001597.s004.doc]

```
CLUSTAL W (1.83) multiple sequence alignment
http://www.ebi.ac.uk/Tools/clustalw/index.html

CKA_H.sapiens_gi|47078276           -HISVIRGGLSNMLFQCSLPDTTATLGDEP--RKVLLRLYGAILQMRSCN
CKA_M.musculus_gi|70908364          -HISVIRGGLSNMLFQCSLPDSIASVGDEP--RKVLLRLYGAILKM----
CKB_H.sapiensÂ¬_gi|133777770        -RVYPVSGGLSNLLFRCSLPDHLPSVGEEP--REVLLRLYGAILQG----
CKB_M.musculus_gi|148672392         -SVCPVSGGLSNLLFRCSLPNHVPSVGGEP--REVLLRLYGAILQG----
CK_C.elegans_pdb_1nw1               -RISRIKGGMSNMLFLCRLSEVYPPIRNEP--NKVLLRVYFNPETE----
APH_O.alexandrii_gi|83859474        -TVEQFKGGQSNPTYKLSTPGAQYVLRRKP--PGKLLPSAHAVDREYR--
APH_M.maris_gi|114569176            -TVEQFKGGQSNPTYKLVTPGKAYVLRRKP--PGKLLPSAHAVDREFR--
APH_B.phytofirmans_gi|11803915      -TLEQFAGGQSNPTFKLLTPSRSYVMRAKPGPAAKLLPSAHAVEREYR--
ACAD10_H.sapiens_ENSP000003251      -ELLQFDHGQSNPTYYIRLANRDLVLRKKP--PGTLLPSAHAIEREFR--
APH_A.cryptum_gi|148261103          -TIRQFQGGQSNPTYLIETAARNYVLRKKP--PGTLLPSAHQIDREFR--
ACAD11_H.sapiens_ENSP000002649      -TIAQYRAGKSNPTFYLQKGFQTYVLRKKP--PGSLLPKAHQIDREFK--
CKB_C.elegans_gi|32565112           -NVSRVHGGQSNHMFHVTSSTS-----ATP----YLLRIHRQPPSQ----
APH_K.pneumoniae_pdb_1ND4           DWAQQTIGCSDAAVFRLSAQGRPVLFVKTD-----LSGALNELQDEAAR-
APH_E.faecalis_pdb_1J7L             RCVKDTEGMSPAKVYKLVGENENLYLKMTD---SRYKGTTYDVEREKD--
                                                  :

CKA_H.sapiens_gi|47078276           KEGSEQAQKENEFQGAEAMVLESVMFAILAERSLGPKLYGIFPQGRLEQF
CKA_M.musculus_gi|70908364          --------------GAEAMVLESVMFAILAERSLGPKLFGIFPQGRLEQF
CKB_H.sapiensÂ¬_gi|133777770        ---------------VDSLVLESVMFAILAERSLGPQLYGVFPEGRLEQY
CKB_M.musculus_gi|148672392         ---------------VDSLVLESVMFAILAERSLGPQLYGVFPEGRLEQY
CK_C.elegans_pdb_1nw1               ----------------SHLVAESVIFTLLSERHLGPKLYGIFSGGRLEEY
APH_O.alexandrii_gi|83859474        ----------------VMKALGEQGFPAPHMFGLCEDDSVIGTAFYIMDF
APH_M.maris_gi|114569176            ----------------VMKALGGAGFPAPVMHGLCMEREVIGTEFYIMDF
APH_B.phytofirmans_gi|11803915      ----------------VMHALADTDVPVAKMLALCEDESVIGRAFYVMEF
ACAD10_H.sapiens_ENSP000003251      ----------------IMKALANAGVPVPNVLDLCEDSSVIGTPFYVMEY
APH_A.cryptum_gi|148261103          ----------------IQCALAATDVPVAPMLHYCTDAAVIGTEFYVMGH
ACAD11_H.sapiens_ENSP000002649      ----------------VQKALFSIGFPVPKPILYCSDTSVIGTEFYVMEH
CKB_C.elegans_gi|32565112           ------------------VFTDTVNLAIFSERGLGPKLYGFFEGGRMEEF
APH_K.pneumoniae_pdb_1ND4           -----------------LSWLATTGVPCAAVLDVVTEA---GRDWLLLGE
APH_E.faecalis_pdb_1J7L             -----------------MMLWLEGKLPVPKVLHFERHD---GWSNLLMSE
                                                             ..         .         :
```

```
CKA_H.sapiens_gi|47078276           IPSRRLDTEELSLPDISAEIAEKMATFHGMKMPFNKE---PKWLFGTMEK
CKA_M.musculus_gi|70908364          IPSRRLDTEELRLPDISAEIAEKMATFHGMKMPFNKE---PKWLFGTMEK
CKB_H.sapiensÂ¬_gi|133777770        IPSRPLKTQELREPVLSAAIATKMAQFHGMEMPFTKE---PHWLFGTMER
CKB_M.musculus_gi|148672392         LPSRPLKTQELRDPVLSGAIATRMARFHGMEMPFTKE---PRWLFGTMER
CK_C.elegans_pdb_1nw1               IPSRPLSCHEISLAHMSTKIAKRVAKVHQLEVPIWKE---PDYLCEALQR
APH_O.alexandrii_gi|83859474        VAGRIFW--DPYLPDLSPDQRGAIYDASNATLAQLHS---IDHEAAGLGD
APH_M.maris_gi|114569176            VEGRIFW--DPLLPDLDKTERGAIYDASNATLAHLHG---IDFEAAGLGD
APH_B.phytofirmans_gi|11803915      VEGRVLW--DQSLPGMTPAERAAIYDEMNRVIAALHS---VDVAAVGLAD
ACAD10_H.sapiens_ENSP000003251      CPGLIYK--DPSLPGLEPSHRRAIYTAMNTVLCKIHS---VDLQAVGLED
APH_A.cryptum_gi|148261103          VAGRVFH--DVMMPGLAPEERGAIHRQIFETIAALHK---VDYNAVGLTG
ACAD11_H.sapiens_ENSP000002649      VQGRIFR--DLTIPGLSPAERSAIYVATVETLAQLRS---LNIQSLQLEG
CKB_C.elegans_gi|32565112           LPSKTFDVNDVLVPENSRKIGAIFPLYHSINVPVSKSRRCVHLMREWLNG
APH_K.pneumoniae_pdb_1ND4           VPG-----QDLLSSHLAPAEKVSIMADAMRRLHTLDP---ATCPFDHQAK
APH_E.faecalis_pdb_1J7L             ADGVLCS--EEYEDEQSPEKIIELYAECIRLFHSIDIS---DCPYTNSLD
                                      .      :             .       .

CKA_H.sapiens_gi|47078276           YLKEVL-RIKFTEESRIKKLH---KLLSYNLPLELENLRSLLESTP-SPV
CKA_M.musculus_gi|70908364          YLNQVL-RLKFSREARVQQLH---KILSYNLPLELENLRSLLQYTR-SPV
CKB_H.sapiensÂ¬_gi|133777770        YLKQIQ-DLPPTG---LPEMN---LLEMYSLKDEMGNLRKLLESTP-SPV
CKB_M.musculus_gi|148672392         YLKQIQ-DLPSTS---LPQMN---LVEMYSLKDEMNSLRKLLDDTP-SPV
CK_C.elegans_pdb_1nw1               WLKQLTGTVDAEHRFDLPEECGVSSVNCLDLARELEFLRAHISLSK-SPV
APH_O.alexandrii_gi|83859474        YGKPGN-YFERSIGRWSKQYKAAETETVAAMDKLIEWLPEHAPAQ--ERT
APH_M.maris_gi|114569176            YGKPGN-YFERQIGRWTKQYRAAETTTVPAMDRLIEWLPTAAPDQ--ERT
APH_B.phytofirmans_gi|11803915      YGKPGN-YFARQIGRWSKQYIASETEPIDAMQRLIEWLPQHMPAETGERA
ACAD10_H.sapiens_ENSP000003251      YGKQGD-YIPRQVRTWVKQYRASETSTIPAMERLIEWLPLHLPRQ--QRT
APH_A.cryptum_gi|148261103          FGRPDH-YVARQIERWTKQYEASKTEDFAPMTDLIAWLRANIPER--DES
ACAD11_H.sapiens_ENSP000002649      YGIGAG-YCKRQVSTWTKQYQAAAHQDIPAMQQLSEWLMKNLPDND-NEE
CKB_C.elegans_gi|32565112           YESLGG-GDYEILPTTVNYSDHPKSVSIKDLNHEIDNFEKWSTEIFEHTL
APH_K.pneumoniae_pdb_1ND4           HRIERA--RTRMEAGLVDQDDLDEEHQGLAPAELFARLKARMPDG--EDL
APH_E.faecalis_pdb_1J7L             SRLAELDYLLNNDLADVDCENWEEDTPFKDPRELYDFLKTEKPEE---EL
                                                                         :
```

```
CKA_H.sapiens_gi|47078276           VFCHNDCQEGNILLLEGRE----------------------NSEKQKLML
CKA_M.musculus_gi|70908364          VFCHNDCQEGNILLLEGQE----------------------NSERRKLML
CKB_H.sapiensÂ¬_gi|133777770        VFCHNDIQEGNILLLSEPE----------------------NAD--SLML
CKB_M.musculus_gi|148672392         VFCHNDIQEGNILLLSEPD----------------------SDD--NLML
CK_C.elegans_pdb_1nw1               TFCHNDLQEGNILLPKASSGNIRMPSLSDETQALGNSLSAFNPADPRLVL
APH_O.alexandrii_gi|83859474        SVVHGDYRLDNMIFHPTEP--------------------------RVIAV
APH_M.maris_gi|114569176            SVVHGDYRLDNMVFHPTEP--------------------------RVIAV
APH_B.phytofirmans_gi|11803915      SVVHGDYRLDNLIFHPEEP--------------------------RVLAV
ACAD10_H.sapiens_ENSP000003251      TVVHGDFRLDNLVFHPEEP--------------------------EVLAV
APH_A.cryptum_gi|148261103          AIVHGDFRLGNMMIHPTEP--------------------------RIVAV
ACAD11_H.sapiens_ENSP000002649      NLIHGDFRLDNIVFHPKEC--------------------------RVIAV
CKB_C.elegans_gi|32565112           VFSHNDLASTNILELNSTK---------------------------ELVL
APH_K.pneumoniae_pdb_1ND4           VVTHGDACLPNIMVENGRFS----------------------------GF
APH_E.faecalis_pdb_1J7L             VFSHGDLGDSNIFVKDGKVS----------------------------GF
                                     . *.*    *:.                                    .

CKA_H.sapiens_gi|47078276           IDFEYSSYNYRGFDIGNHFCEWMYDY----SYEKYPFFRANIRKYPTKKQ
CKA_M.musculus_gi|70908364          IDFEYSSYNYRGFDIGNHFCEWMYDY----TYEKYPFFRANIQKYPSRKQ
CKB_H.sapiensÂ¬_gi|133777770        VDFEYSSYNYRGFDIGNHFCEWVYDY----THEEWPFYKARPTDYPTQEQ
CKB_M.musculus_gi|148672392         VDFEYSSYNYRGFDIGNHFCEWVYDY----TYEEWPFYKARPTDYPTREQ
CK_C.elegans_pdb_1nw1               IDFEYASYNYRAFDFANHFIEWTIDY----DIDEAPFYKIQTENFPENDQ
APH_O.alexandrii_gi|83859474        LDWELSTLGDPLADFTYQLMQWRTPK---DIRNGFLGQDLKAMGIPTEEE
APH_M.maris_gi|114569176            LDWELSTLGDPLADFTYQLMGWVMPP---EIRNGLLGVDIASLGIPDIEQ
APH_B.phytofirmans_gi|11803915      LDWELSTLGDPLADFAYHCMAWHVDP---AQFRGIAGLDWVTLGIPDEQQ
ACAD10_H.sapiens_ENSP000003251      LDWELSTLGDPLADVAYSCLAHYLPSSF-PVLRGINDCDLTQLGIPAAEE
APH_A.cryptum_gi|148261103          LDWELSTIGHPLADLAYCCMSYHLPPGSGPAMAGYQGVDLAELGLMSEAE
ACAD11_H.sapiens_ENSP000002649      LDWELSTIGHPLSDLAHFSLFYFWPR---TVPMINQGSYSENSGIPSMEE
CKB_C.elegans_gi|32565112           IDWEFGTYNWRGFDLAMHLSETAIDY----RVPFPPGIKMNGDLIDNPPN
APH_K.pneumoniae_pdb_1ND4           IDCGRLGVADRYQDIALATRDIAEELGGEWADRFLVLYGIAAPDSQRIAF
APH_E.faecalis_pdb_1J7L             IDLGRSGRADKWYDIAFCVRSIREDIGEEQYVELFFDLLGIKPDWEKIKY
                                    :*           *.
```

```
CKA_H.sapiens_gi|47078276          ------------------
CKA_M.musculus_gi|70908364         ------------------
CKB_H.sapiensÂ¬_gi|133777770       ------------------
CKB_M.musculus_gi|148672392        ------------------
CK_C.elegans_pdb_1nw1              ------------------
APH_O.alexandrii_gi|83859474       YVKAYCERTGRNGIPKLD
APH_M.maris_gi|114569176           Y-----------------
APH_B.phytofirmans_gi|11803915     Y-----------------
ACAD10_H.sapiens_ENSP000003251     Y-----------------
APH_A.cryptum_gi|148261103         AL----------------
ACAD11_H.sapiens_ENSP000002649     L-----------------
CKB_C.elegans_gi|32565112          ------------------
APH_K.pneumoniae_pdb_1ND4          Y-----------------
APH_E.faecalis_pdb_1J7L            Y-----------------
```

Supplementary Figure S1
